# A Deep Learning-Based Model for the Automated Assessment of the Activity of a Single Worker

**DOI:** 10.3390/s20092571

**Published:** 2020-04-30

**Authors:** Justyna Patalas-Maliszewska, Daniel Halikowski

**Affiliations:** 1 Institute of Mechanical Engineering, University of Zielona Góra, 65-417 Zielona Góra, Poland; 2 Faculty of Technical Science, University of Applied Science in Nysa, 48-300 Nysa, Poland; daniel.halikowski@pwsz.nysa.pl

**Keywords:** assessment of the activity of a single worker, deep learning, convolution neural network (CNN), regions with convolutional neural networks (R-CNN), recognition of employee activity, object detector, verification

## Abstract

Nowadays, it is necessary to verify the accuracy of servicing work, undertaken by new employees, within a manufacturing company. A gap in the research has been observed in effective methods to automatically evaluate the work of a newly employed worker. The main purpose of the study is to build a new, deep learning model, in order to automatically assess the activity of the single worker. The proposed approach integrates the methods known as CNN, CNN + SVM, CNN + R-CNN, four new algorithms and a piece of work from a selected company, using this as an own-created dataset, in order to create a solution enabling assessment of the activity of single workers. Data were collected from an operational manufacturing cell without any guided or scripted work. The results reveal that the model developed is able to accurately detect the correctness of the work process. The model’s accuracy mostly exceeds current state-of-the-art methods for detecting work activities in manufacturing. The proposed two-stage approach, firstly, assigning the appropriate graphic instruction to a given employee’s activity using CNN and then using R-CNN to isolate the object from the reference frames, yields 94.01% and 73.15% accuracy of identification, respectively.

## 1. Introduction

These days, due to the need both to improve worker productivity and product quality within manufacturing companies, managers must introduce more automated work assistance and training systems for operational tasks undertaken by workers. Moreover, a current problem in production enterprises is the continuous turnover of employees, in particular, the turnover of specialists; therefore, it is necessary to conduct specialist training for new employees and also verify their skills. The training process is always time-consuming and also requires the involvement of experienced employees. Our motivation for undertaking this research is the development of an effective tool, the use of which will improve the efficiency of the training process for new employees in manufacturing companies in the context of reducing costs and in the time spent by enterprises. This is a particularly important problem in the context of undertaking specialist servicing work at a given workplace. So, the research problem was formulated as the search for a scenario for learning new work activities, which is built automatically and is based on the specialist knowledge available, regarding the performance of a given work activity without the requirement to involve experts.

The automated assessment and measurement of the activity of workers is essential for the rapid and precise diagnosis of quality work, especially in the training process of the new employee. Even with a video system in place, it is nearly impossible to quickly find and access recordings showing actions being incorrectly carried out. 

Several research studies have been conducted that implement classifiers such as the support vector machine (SVM), principal component analysis (PCA), linear discriminant analysis (LDA), random forest, K-nearest neighbours, the hidden Markov model [1,2] and deep learning algorithms [3,4,5,6,7,8,9,10] to develop recognition of human activity (HAR); these research studies have achieved promising results; see [11] for a more comprehensive overview. Deep learning is also used, successfully, in object and video recognition [12] and has the ability to automatically learn from unlabelled, raw, sensor data [13]. The process of image recognition and analysis is closely related to the process of video analysis, understood as a sequence of individual images. The probability of belonging to a class is calculated on the basis of the features extracted from individual frames [14].

The convolutional neural network (CNN) achieves remarkable successes in many tasks such as image and video recognition [15]. The architecture of the CNN network is a system of connected processing units, or neurons, whose output data are input data for subsequent processing units [16]. Each of the neurons has a weight *W = (w_1_*, *w_2_*,*…*, *w_n_)* where *n ϵ* N, input vector with the same dimension *X = (x_1_*, *x_2_*,*…*, *x_n_)* where *n ϵ* N and threshold or bias. Processing units are grouped into layers, creating subsequent layers (e.g., convolutional). This sort of network relies on the natural, stationary property of an image, i.e., the statistics of one part of the image are the same as any other part and information extracted at one part of the image can also be employed in other parts [14]. 

The use of CNNs in the video processing field, in applications useful to industry, is still significant and growing [17]. CNN approaches are capable of monitoring laser-based manufacturing processes [17,18,19,20]. However, it is difficult to find solutions that meet the automated assessment and measurement requirements of the activity of the single worker. Therefore, we discuss recent studies for the implementation of a discriminative, deep learning method for recognising human activity, based on data from various sensors, in the context of carrying out and assessing work. Ding et al. [12] proposed a model for recognising the unsafe actions of workers, using a convolution neural network (CNN) and long, short-term memory (LSTM). Sharma et al. [21] also used the LSTM-based, deep learning technique to obtain emotion variations, based on the data from EEG signals. Rude et al. [22] investigated the feasibility of using hidden Markov models and naive Bayes K-means for recognising worker activity in manufacturing processes using the Kinect sensor. Sathyanarayana et al. [23] proposed the use of a CNN to predict the relationship between physical activities and sleep patterns using accelerometer and gyroscope sensors. Zeng et al., [24] proposed an approach to automatically extract discriminative features for recognising activity, based on CNN and based on the data from the mobile sensors embedded in smart phones. Jaouedi et al. [25] presented an approach based on the analysis of video content where features are based on all visual characteristic of each frame of a video sequence, using the recurrent neural networks model with the gated recurrent unit. 

Our approach, as presented, integrates SVM, a convolutional neural network (CNN) and regions with convolutional neural networks (R-CNN) along with four new algorithms, to automatically verify the work being carried out by a new worker in a company. In the first stage of our model, video cameras are used to collect motion data from the workplace. At the same time, a graphic instructions database for this workplace is being built, based on the reference video from the workplace. Next, the reference frames and objects are determined and the characteristics of these frames, using respectively CNN with SVM and CNN with R-CNN, are extracted from reference video material. In the next stage, the reference frames are determined, in accordance with the subsequent stages of the work and are recorded, in a sample video; the dataset is created also. In the next stage, the work carried out by a new employee was compared (tested video sequence) to the previously designated reference frames, using CNN. The features and objects of the test material frames were then compared with the work database previously created. Based on the results of the comparison, irregularities in tasks undertaken at a workplace are immediately detected and the instruction is no longer displayed.

An experiment is implemented to demonstrate the effectiveness of the proposed model developed, which enables assessment of the completed work. 

Our model is evaluated on a self-created dataset, containing 3029 files of which 4426 files are regions of interest (ROIs). The 3029 files are frames of standard video material containing servicing work, carried out in a given position. Data were collected from an operational manufacturing cell without any guided or scripted work. Each frame contains objects that had to be selected before the R-CNN training (ROI).

A novel aspect of this study is that it is based on live data collected from an operational manufacturing cell without any guided or scripted work and also its two-stage approach, firstly, assigning the appropriate graphic instruction to a given employee’s activity, using CNN and then using R-CNN to isolate the object from the reference frames and evaluation of the model. The major contribution of the work is as follows: (1) extraction of the characteristics of the reference frames, based on analysis of the video content and representation of a piece of work from a selected company image, using CNN with SVM; (2) the preparation of a workplace image database, which is not publicly available; (3) comparison of the features and objects in the frames of test material with the database of the work previously created, as well as comparing them with their features and objects, using CNN and R-CNN; (4) experimental results and analysis of the proposed approach. The proposed, two-stage approach, firstly, assigning the appropriate graphic instruction to a given employee’s activity using CNN, and then using R-CNN to isolate the object from the reference frames yields 94.01% and 73.15% identification accuracy, respectively. 

## 2. Related Works and Motivation

A current problem in manufacturing companies is the continuous turnover of employees, in particular specialists, and therefore it is necessary to conduct specialist training for new employees and also to verify their skills. There is a lack of solutions on the market for the automatic supervision of work to be carried out in the production workplace. 

The framework presented in this paper consists of a deep-learning based approach for assessing the work activity of a new, single worker, manufacturing cell, based on automatically generated instruction. The research niche of studies on HAR systems as part of a framework to enable the continuous monitoring of worker activity in a manufacturing company is identified, because developed HAR solutions are usually for the area of ambient assisted living, the detection of sports injuries, care of the elderly, rehabilitation and smart home environments [13]. Two types of HAR can be distinguished: video-based HAR and sensor-based HAR [26]. Video-based HAR analyses videos or images, while sensor-based HAR focuses on the data from an accelerometer, gyroscope, Bluetooth or mobile sensors [7]. In our approach, work in a given workplace is recorded and constitutes reference video material. 

To assess each servicing operation being carried out by a new employee, it is necessary to extract the reference feature and the reference object from the data—the reference video sequence—in order to compare the features and objects of the test material frames, that is, the video sequence tested. Therefore, it was also necessary to integrate CNN with classifiers in our approach. Various research efforts have been geared toward obtaining robust and effective features by combining CNN and classifiers. 

CNN with SVM is used to diagnose diseases [27,28,29], diagnostics of automotive components, image recognition [20,28], image classification [29], facial recognition, target detection [28], recognition of vehicle colours [30], stock market forecasts [31] and the recognition of animal species [32]. Knowing that monitoring the time series data collected is not an easy task, symbolic aggregate approximation (SAX) for data processing can be applied [33]. SAX is used to discretise time series data [34]. It is also used to detect anomalies, analyse differences in sets and visualise bitmaps, using time series grouping, based on the compression of distance measurement [35]. SAX is also used to search for rules in time series, find motives of indefinite length, find repetitive patterns in robot sensors, make space-time connections in trajectories and match 2D shapes [35]. SAX efficiently reduces high dimensional time series data for application in various types of data mining algorithms [36]; however, SAX cannot complete feature extraction tasks [36]. Huang and Lu [37] proposed an algorithm combining dynamic time warping (DTW) and compressed learning (CL) techniques for the classification of temporal data. DTW is used as a measure of distance and can also be used as a data filter for network convolution layers, in order to create more efficient feature extractors for CNN [38,39]. It is used in speech recognition, human motion animation, human activity and in time series classification [40]. DTW and artificial neural networks are also integrated into the model for predicting remaining useful life [41]. DTW’s computational demand for finding the optimal time alignment path is usually massive, however, thus restricting its use in establishing monitoring systems in real time [37]. CNN with long, short-term memory (LSTM) is used for such tasks as handwriting recognition [42], speech sequence processing [42,43,44,45], anomaly detection in network traffic [46], time series processing [47] and prediction [44], traffic forecasting [47], video sequence analysis [48,49], image labelling [42], binarisation and texture analysis [42]. LSTM is also used to analyse images [43] and objects therein [42,49]. In the literature, one comes across the use of this architecture with reference to pedestrian modelling, when analysing individual body parts [43]. In addition, LSTM also has great potential in image classification and object detection by modelling spatial dependencies and analysing contextual information [43]. 

However, as detailed above, there are many examples of CNN applications in the HAR area (see [13] for a more comprehensive overview) and of CNN combined with classifiers, but no solutions were found concerning working at a given workplace in manufacturing (Table 1). In the context of verifying the skills of the new employee, we did not find any sources that presented the use of these approaches for assessing work, based on pre-recorded video material.

Our approach is closest to the related model [22], but we monitor single worker activity in manufacturing, using video and we use CNN combined with classifiers: CNN + SVM and CNN + R-CNN. 

We agree with [13], that areas that require further research should combine expert knowledge with a deep learning algorithm and different, unsupervised feature learning methods. In order therefore, to assess and control the activity undertaken by a new worker, based on pre-recorded video material and on workstation instruction, we apply CNN, SVM, and R-CNN. R-CNN is an object detection framework to classify image regions within an image [50]. The deep learning model presented includes, on the one hand, the collection and formalisation of specialist knowledge in the company, while including, on the other hand, support for the control process. We believe that the data we present in this paper are unique, because the data were collected in a real-workplace with a camera and include tasks of a piece of work from a selected company, namely, the repair of a solid-fuel boiler.

## 3. Model and Methods 

As mentioned earlier, our idea is to create a model for verifying the skills of a new employee to undertake servicing work, namely, the repair of a solid-fuel using boiler. We believe that the supervision and control of this activity can be done automatically, having registered video material containing correct servicing methods, as demonstrated by a specialist.

**Figure 1:**
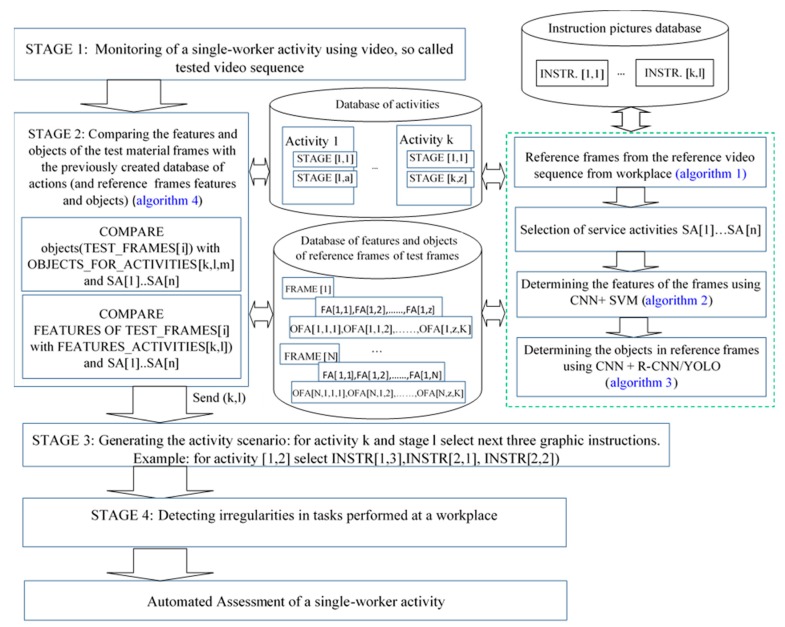
A model for automatically assessing the activity of a single worker, displayed on the basis of the stage of activities identified. Here *i*—number of test frames, *i* ϵ N, *k*—number of servicing procedures stage, *k* ϵ N, *l*—number of stages in a single activity, *l* ϵ N; *m*—number of objects in the frame, *m* ϵ N; TEST_FRAMES—an array storing frames of the video material analysed; FEATURES_ACTIVITIES—jagged array containing the features of the reference frames; OBJECTS_FOR_ACTIVITIES—a jagged array containing object labels for each activity and reference frame; ACTIVITIES—a jagged array storing information of activities and stages of activity; STAGE—activity stage, for example STAGE [1,2] means activity 1 and stage 2; ACTIVITY—servicing procedure activity.

The proposed model consists of the following elements: (1) determining the reference frames and extracting the characteristics of the frames from the reference video material, using CNN with SVM; (2) determining frames of video material, (‘reference frames’ so called) in accordance with the subsequent stages of the servicing work, recorded in a sample video; (3) creating own dataset, (4) assigning the reference frames to each servicing operation: algorithm 1; (5) comparison of the servicing work done by a new employee via a new video recording, to the previously designated reference frames using a CNN trained via ‘a self-created’ dataset: algorithm 2; (6) comparing the features and objects of the test material frames with the database of servicing work, previously created, as well as comparing them with their features and objects using CNN and R-CNN: algorithm 3 and algorithm 4; (7) assessment of the servicing work carried out (Figure 1). 

The elements of the model (Figure 1) highlighted in green are discussed in detail in [11]. In this article, CNN was pledged using ROI and YOLOv3. The model elements that allow the extraction of image features and also the detection of objects, or classes, in the image, were presented in special detail. The research results presented, relate to a two-step approach to the topic of recognising the content found in video frames. In addition to the similarity of frames, the occurrence of objects in these frames was also analysed. The proposed model (Figure 1) is described according to the four new algorithms, developed in order to assess the skills of a new employee, carrying out servicing work automatically.

Algorithm 1: Algorithm for the Assignment of Reference Frames to Servicing Tasks

The creation of a system for automatically assessing the skills of a new employee to undertake servicing work will require partial human intervention at certain stages of the operation. The video sequence containing the correctly registered oven inspection procedure will be analysed by a qualified technician, whose task will be to determine the key stages for each servicing activity carried out in the procedure. The result will be a set of key frames for each stage of the activity; this set will be referred to as the ‘set of reference frames’. The collection can be represented as follows:

*FRM = {FRAME [1], FRAME [2],…,FRAME [n]}*, (1)

where: *FRM*—set of reference frames selected manually by the employee and *n*—the reference frame number, *n* ϵ N.

The set of reference frames, prepared in this way, will be processed by the algorithm which assigns the reference frames to the servicing operations, hereinafter referred to as Algorithm 1. The task of Algorithm 1 will be to analyse the set of FRM frames and divide this set into components for the servicing procedure. A START/STOP rule has been defined according to which, the set of reference frames will be divided into several subsets, each subset having a separate servicing activity. This rule is the occurrence, in the frame set, from which the technical worker and the tool board will be identified. Algorithm 1 performs this task as follows:
**Algorithm 1—Pseudocode:**initialisation;*k*, *l*←0;size (ACTIVITIES [][])←[*k*,*l*]; for *i* = 1 to size(FRM) do   begin      if (compare (FRAME[*i*], REF_FRAME_START_STOP) = true then       begin        inc(*k*);        *l*←0;       end      else       begin        inc(*l*);        size (ACTIVITIES [] []) ← [*k*,*l*];        ACTIVITIES[*k*][*l*] = FRAME[*i*];       end;   end;send→(ACTIVITIES [][]);
where *k*, *l*—are array indexes, *i*—a loop counter, ACTIVITIES—a jagged array storing reference frames for a given activity, FRAME—an array containing all frames, selected manually by the employee and *k*, *l*, *i* ϵ N

The result of Algorithm 1 will be a jagged table containing reference frames for the activities extracted in the servicing work. A table showing the activities and the stages of each activity appears as follows:

(2) $$ACTIVITIES=\left\{\begin{array}{c} A\left[1,1\right],A\left[1,2],\ldots ,A[1,a\right]\\ A\left[2,1],A[2,2],\ldots ,A[1,b\right]\\ A\left[3,1],A[3,2],\ldots ,A[1,c\right]\\ \ldots \\ A\left[N,1],A[N,2],\ldots ,A[N,z\right] \end{array}\right\},$$

where: ACTIVITIES—jagged table containing reference frames assigned to the actions extracted using *Algorithm 1*, A[N,z]—array element for stage z and N-th activity, N—the number of procedure stages, *a*,*…*,*z*—the size of the set of reference frames for a single activity and *N*, *a…z* ϵ N.

Algorithm 2: Algorithm for Determining the Features of the Frames

**Figure 2:**
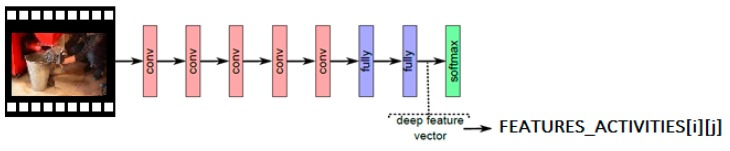
The idea of extracting a feature vector for a frame.

It was assumed that the process of identifying the servicing procedure stage, currently being undertaken by a technical employee, would be based on a comparison of reference frames determined on the basis of the video material of the model and the frames from the video material, on which the sequences carried out by the trained employee were recorded. To do this, the frames determined, by Algorithm 1, stored in the ACTIVITIES table must undergo further processing. For reference frames and for each frame with recorded activities of a technical employee, sets of features describing each frame will be determined using CNN. At a later stage, these features will be compared and classified using the SVM classifier. In addition, the results of comparing the cage features will be the basis for the decision to move on to the next stage of the servicing procedure. The idea of extracting features for the frame *i*, *j* of the FEATURES_ACTIVITIES table, is shown in Figure 2.

Features are extracted using the ALGORITHM FOR DETERMINING FRAME FEATURES, hereinafter referred to as Algorithm 2.
**Algorithm 2—Pseudocode:**initialisation;size (FEATURES_ACTIVITIES [][])←[size(ACTIVITIES[]),size(ACTIVITIES[][])];for *i* = 1 to size (ACTIVITIES []) dobeginfor *j* = 1 to size(ACTIVITIES[*i*][]) do   begin     FEATURES_ACTIVITIES[*i*][*j*] = features(ACTIVITIES[*i*][*j*]));   end;end;
where: *i*, *j*—loop counters, ACTIVITIES—a jagged array storing frames of all activities, FEATURES_ACTIVITIES—a jagged array containing the characteristics of reference frames, features—function that extracts features from the CNN network and *i*, *j* ϵ N

The result of the operation of Algorithm 2 will be a jagged table containing the characteristics of reference frames for the activities extracted in the servicing procedure, as well as the activity stages. 

The array representing the features of each frame, denoting a particular stage of the servicing procedure, is as follows:

(3) $$FEATURES\_ACTIVITIES=\left\{\begin{array}{c} FA\left[1,1],FA[1,2],\ldots \ldots ,FA[1,a\right]\\ FA\left[2,1],FA[2,2],\ldots \ldots ,FA[1,b\right]\\ FA\left[3,1],FA[3,2],\ldots \ldots ,FA[1,c\right]\\ \ldots \\ FA\left[N,1],FA[N,2],\ldots \ldots ,FA[N,z\right] \end{array}\right\}$$

where: *FEATURES_ACTIVITIES*—a jagged array containing the features of each reference frame, *FA[N*,*z]*—array element for frame *z* and *N*-th activity, *N*—the number of instructional sets for all stages of a procedure, *a*,*…z* –the number of frames per stage of the procedure. Each servicing activity has a different number of instructions, depending on the number of frames, therefore it is a jagged board and thus has a different size in the second dimension and *N*, *a…z* ϵ N.

Algorithm 3: Algorithm for Determining Objects from the Reference Frames

In the next stage of the procedure, that is, for each frame of training material depicting the activities being carried out by a technical employee, the features of the image will also be extracted. They will be compared with elements of the FEATURES_ACTIVITIES array and, on this basis, the stage of the current activity will be identified. An additional element supporting the process of identifying the servicing activity, is that of determining the additional set of data associated with the reference frames. This set will store information about objects in reference frames. The objects for each reference frame will be extracted using the algorithm for determining objects from the reference frames, hereinafter referred to as Algorithm 3.
**Algorithm 3—Pseudocode:**initialisation;size (OBJECT_FOR_ACTIVITIES)←[size(ACTIVITIES[]),0,0];for *i* = 1 to size (ACTIVITIES[]) do  begin   for *j* = 1 to size(ACTIVITIES[*i*][]) do     begin      z[] = extract_objects (ACTIVITIES[*i*][*j*]); size (OBJECTS_FOR_ACTIVITIES [][][])←[size(ACTIVITIES[]), size(ACTIVITIES[*i*][]), z];      put_object_labels(OBJECTS_FOR_ACTIVITIES[*i*][*j*][*z*]);      end;  end;
where: *i*, *j*—loop counters, *z*—the collection into which object labels will be extracted, ACTIVITIES—a jagged array storing activity frames; OBJECTS_FOR_ACTIVITIES—a jagged array containing object labels for each reference frame (and activity); *i*, *j* ϵ N.

The result of the above algorithm is a jagged array OBJECTS_FOR_ACTIVITIES, containing object labels for each reference frame. An ex ample of an array of elements for activity [2,1] appears like this:

OBJECTS_FOR_ACTIVITIES [2,1,1] = “SOLID FUEL BOILER”
OBJECTS_FOR_ACTIVITIES [2,1,2] = “MOTOREDUCTOR”

As it is a three-dimensional array, it has not been presented graphically in the article.

Algorithm 4: Algorithm comparing the features and objects of the test material frames with the previously created database of actions (and their features and objects)

This algorithm compares the features and objects of the test material frames with the database of activities previously created, as well as with their features and objects. Having the features of reference frames and information about objects, it will be possible to identify activity stages by comparing the object labels and features of the training material frames, as well as the features and labels of objects for reference frames. This task will enable the algorithm to compare the features and objects of the test material frames with the database of actions previously created, along with their features and objects; this algorithm will henceforth be referred to as Algorithm 4.
**Algorithm 4—pseudocode:**initialisation;size(matching [][])←[size(TEST_FRAMES),2];for *i* = 1 to size (TEST_FRAMES) do  begin    for *k* = 1 to size(FEATURES_ACTIVITIES[]) to     begin      for *l =* 1 to size(FEATURES_ACTIVITIES[*k*][]) do       begin         if (compare(features(TEST_FRAMES[*i*]),FEATURES_ACTIVITIES[*k*,*l*]) *=* true) then           begin            for *m =* 1 to size(OBJECTS_FOR_ACTIVITIES[*k*][*l*][]) to             begin                If objects(TEST_FRAMES[*i*]) *=* OBJECTS_FOR_                  ACTIVITIES[*k*,*l*,*m*]) then                begin                  matching[*i*][0] *= k*;                  matching[*i*][1] *= l*;                  send→ (*k*,*l*);                end;             end;           end;         end;     end;send→ (matching[][])
where: *i*, *k*, *l*, *m* –loop counters, matching [][]—is an array that stores information about the indexes of the matched reference frame, TEST_FRAMES—an array storing frames of the video material analysed, FEATURES_ACTIVITIES—jagged array containing the features of reference frames, OBJECTS_FOR_ACTIVITIES—a jagged array containing object labels for each activity and reference frame and *k*, *l*, *m*, *i* ϵ N.

As a result of the algorithm, indexes k and l were obtained; these determine the current stage of the servicing procedure and will be used in the process, to generate the procedure’s scenario, that is, to generate a set of instructions for displaying on a screen. 

The proposed approach enables the automated assessment of the activity of a single worker, based on finding differences or irregularities in each activity, done in relation to the reference video material. The model (Figure 1) was implemented for the servicing of a solid fuel boiler: “Repair of a solid fuel boiler”, conducted in Matlab 2019 and presented below as a description of the case.

As part of the research, a sample video of about 57 s was prepared and processed into single frames (30 frames per second) and totalling 1727 pieces. The set also contained individual frames that are reference frames START_STOP. 

In the next step, reference frames were manually determined; the activities and stages for each activity were then determined. This was done using Algorithm 1 which is the algorithm used to assign reference frames to servicing operations. The assumption was to prepare the material for the following stages of servicing work: [1] STOPPING THE SOLID FUEL BOILER, [2] UNSCREWING THE MOTOREDUCTOR SCREWS, [3] REMOVING THE CLEANER, [4] REMOVING THE MOTOREDUCTOR WITH THE AUGER, [5] ASSEMBLING THE AUGER [6] TURNING THE MOTOREDUCTOR MOUNTING SCREWS, [7] INSTALLING THE CLEANER.

The table of elements show the activities and reference frames associated with each stage of the activity (Table 2).

Each action was preceded by a START_STOP frame, as, also, was the end of the servicing. The number of activity stages with START_STOP frames is 30. The designated reference frames were processed in the CNN network where the characteristics of each frame were captured and a 30 × 512 size table was created, containing the data obtained about the characteristics, using Algorithm 2, the algorithm which determines the features of the frames. Feature values for better display have been multiplied by 100.

All 1727 frames of the test material were then passed through the network. The features, thus obtained, were recorded in a table, size 1727 × 512. Feature values for better display have also been multiplied by 100, using Algorithm 2 also, the algorithm which determines the features of the frames.

The next step was to prepare a data structure, to store information about the activity stages and the activities detected. For this purpose, an active table was prepared, which appears as:

*Active* = [0 0 0 −1;1 1 0 −1;1 2 0 −1;1 3 0 −1;0 0 0 −1;2 1 0 −1;2 2 0 −1;2 3 0 −1;0 0 0 −1;3 1 0 −1;3 2 0 −1;3 3 0 −1;3 4 0 −1;0 0 0 −1;4 1 0 −1;4 2 0 −1;4 3 0 −1;4 4 0 −1;0 0 0 −1;5 1 0 −1;5 2 0 −1;5 3 0 −1;0 0 0 −1;6 1 0 −1;6 2 0 −1;6 3 0 −1;0 0 0 −1;7 1 0 −1;7 2 0 −1;0 0 0 −1];

Elements [0 0 0 −1] are data for the frame START_STOP. The other elements indicate the stages of the activity. For example [2 1 0 −1] means the second activity, the first stage. The remaining elements in the record mean: no stage occurrence (0—none, 1 occurrence) and the test frame found (−1 is no hit).

Next we consider Algorithm 4. This algorithm compares the features and objects of the test material frames with the database of actions previously created, along with their features and objects; it was used to compare the features of each reference frame with the frame of the test video. Matlab’s small function was also used for this. Values indicating differences in the characteristics of individual reference and test frames are placed in a 30 × 1727 sized table. Algorithm 4 was also used to determine the stage of activities based on the similarity of the individual reference frame and the test frames (Table 3). 

The limit value for the difference between test and reference frames can be adjusted in the algorithm which compares reference and test frames. Currently, this value is 9.9921. The value was calculated, based on the average of the sum of the differences between the individual frames, with respect to reference frames.

## 4. Research Results

Our model was evaluated in two steps. In the first step, the accuracy of the model was checked in relation to the feature frames (1727 pieces), i.e., compliance of the reference frames with the test material frames was calculated (Table 4). In this model, we used resnet18 network architecture. The values in the "compatibility in%" column indicate the percentage of compliance of the features analysed. As already mentioned, based on these values, part of the process of identifying the activity, undertaken by the employee, takes place. The percentage value of each feature of the individual test and reference frames was calculated and then the average similarity value of the test material frame to the reference material frame was obtained at 94.0098%.

The second step in the model’s evaluation includes objects appearing in frames. The algorithm that extends objects appearing on the frames of the video material is implemented on the dataset contained 3029 files in which are 4426 ROIs (region of interest). Due to the fact that the model’s operation is based on objects specific to a given field, it was impossible to train the CNN network on a ready set of learning data. The available datasets do not contain class objects corresponding to the objects appearing on the video material analysed: Wrench, Motoreductor, Furnace, Hand, Bucket, Burner auger, Cuvette, Controller. Therefore, an own dataset, with designated ROIs, was created. For this reason, the dataset file only has 3029 files. The marked ROI areas contained objects of the following classes: Wrench, Motoreductor, Furnace, Hand, Bucket, Burner auger, Cuvette, Controller.

For further research—and in order to confirm the effectiveness of our proposed approach—the “person” object was tested based on the publicly available COCO dataset. Yolov3 detected 100% of objects out of 216 frames (or 216 images) containing the “person”. However, trained by own created dataset, identification accuracy yields 73.15%. Therefore the own created dataset was adopted for further research as it seems that the identification accuracy obtained is satisfactory.

In our article, we conducted the research experiments in which we used three types of CNN networks. The first network was based on resnet18 (with SVM) and was used to extract frame features. The frame features are one of the key elements deciding whether the activity performed by the new employed worker is correct. For object detection with R-CNN detector we user Cifar10Net architecture. For real-time image analysis, a model based on Yolov3 (COCO) was created. We decided to use the Resnet18 network in the process of distinguishing features due to better efficiency in their matching (Table 5). 

Next it is necessary to check the classes of objects found on the frames of the video material. For this purpose, a combination of CNN network and R-CNN detector was used. In this case, the network was based on the Cifar10Net architecture. 

We decided to use the Cifar10Net network in the process of object detection due to better efficiency object recognition. It is the basic network model used to classify the CIFAR 10 dataset and is often used in image recognition techniques [51,52]. For Cifar10Net, objects on 37 of 60 files were detected, for AlexNet, objects on 29 of 60 files were detected (Table 6).

We also compared the results of object detection using Cifar10Net and AlexNet with the results of object detection using YOLOv3 (Table 7).

The experiment was carried out in Matlab R2019a. Considering network performance, we decided that research experiments would be conducted on a cifar10Net based network architecture model. The neural network used in Matlab had the following layers:

(1) 1 × 1 ImageInputLayer
(2) 1 × 1 Convolution2DLayer
(3) 1 × 1 ReLULayer
(4) 1 × 1 MaxPooling2DLayer
(5) 1 × 1 Convolution2DLayer
(6) 1 × 1 ReLULayer
(7) 1 × 1 MaxPooling2DLayer
(8) 1 × 1 Convolution2DLayer
(9) 1 × 1 ReLULayer
(10) 1 × 1 MaxPooling2DLayer
(11) 1 × 1 FullyConnectedLayer
(12) 1 × 1 ReLULayer
(13) 1 × 1 FullyConnectedLayer
(14) 1 × 1 SoftmaxLayer
(15) 1 × 1 ClassificationOutputlayer

With the help of Algorithm 4 (the algorithm comparing the features and objects of the test material frames with the previously created database of actions and their features and objects), frames from the test material were determined for individual reference frames. Frames were determined, whose features were similar to those of reference frames; the features were compared using the same function of the Matlab R2019a programme. In addition, with the help of Algorithm 3 (the algorithm for determining objects from reference frames), the objects located in these frames were extracted for each reference and test frame. This was possible thanks to the trained CNN and R-CNN networks, i.e., an object detector using a previously trained convolution network.

Based on the teaching set containing 3029 files, a convolution network was trained to find objects of the following classes by R-CNN: Wrench, Motoreductor, Furnace, Hand, Bucket, Burner auger, Cuvette, Controller.

A trained convection network, together with an object detector based on it, was able to detect objects of the following classes for specially prepared graphic files: Gearmotor, Furnace, Hand, Bucket, Snail, Cuvette, Controller (8/8); unfortunately the network was only once able to identify the Key object. ROI for the objects in these files were separated from each other and the objects contained in the images, corresponded to the shape and form of the objects contained in the neural network training set. Thanks to this, for specially prepared files, an efficiency object detection rate of 100%, by the neural network, was obtained (Table 8).

With regard to the reference frames and reference objects, the use of CNN and CNN + R-CNN enables a “similar” frame and object to be found; the value for the similarity criterion can be adjusted; finding and determining the stage of the activity, based on the similarity of the reference and the test frames and objects, since each test frame and test object is used only once, in order that one can be sure of the correct order of the work stages identified. However, only the re-checking and comparison of the material received, with the reference material, using R-CNN, ensures accuracy in the detection of mistakes in the activities carried out by the workers.

## 5. Discussion

The training process within a manufacturing company is always time-consuming and also requires the involvement of experienced employees. Our motivation for undertaking this research was the development of an effective tool, the use of which will improve efficiency in assessing the activity of a new, single worker in manufacturing companies in the context of reducing costs and time. This is a particularly important problem in the context of the undertaking of specialist, servicing work at a given workplace. We have developed a useful model for verifying the skills of a new employee, displayed on the basis of the stage of those activities identified and on an indication of the errors in the work carried out; this integrates CNN and R-CNN and also a network of four new algorithms: an algorithm to assign reference frames to servicing operations; an algorithm to determine the features of the frames; an algorithm to determine the objects in the reference frames and an algorithm to compare the features and objects of the test material frames with the database of actions—and their features and objects—as previously created. 

The efficacy of the deep learning approach proposed was computed in two steps. In the first step, the accuracy of the model was checked in relation to the feature frames; identification accuracy of 94.01% was achieved. In the second step a trained convolutional network, together with an object detector, based on it was able to detect objects of the following classes for specially prepared graphic files; 73.15% efficiency of object detection was obtained. The model’s accuracy mostly exceeds current state-of-the-art methods for detecting work activities in manufacturing. The comparison of results with existing state-of-the-art methods was made on the basis of the sources collected and discussed in Table 1 (Table 9).

Ding et al. [12] employed CNN + LSTM to automatically recognise workers’ unsafe actions. The model’s performance was compared with four types of feature descriptor: the histogram of oriented gradients (HOG), the histogram of optical flow (HOF) and motion boundary histograms (MBH). They obtained a 97% classification accuracy for safe actions and 92% for unsafe actions. Zhong et al. [28] used CNN networks to diagnose faults in a gas turbine. The accuracy of their model was 93.44%. Zhang et al. [36] used CNN networks for intelligent machine diagnosis. The accuracy of their model was 88% showing the advantage of their solution (ISAX) over RMS. The person identification system was presented in the article by Bai et al. [44]. The effectiveness of their solution using CNN + LSTM was about 70%, depending on the technology. Work related to the automated system for the recognition of emotions, based on higher-order statistics and the deep learning algorithm was presented by Sharma et al. [21]. Their solution reached an average of around 87%. The work of Jaouedi et al [25] is also interesting. They presented a new, hybrid, deep learning model for the recognition of human activity. The results they achieve are very promising and generally remain above 85%, with one exception. Rude et al. [22] in their work present the recognition of tasks from joint tracking data in an operational manufacturing cell. 

The research most similar to the proposed model is the solution proposed by Tao et al. [5]. They proposed an employee training system using armbands. The effectiveness of this solution, according to the information contained in the article, is as much as 97%. However our approach refers to the process of creating workplace instructions using a two-step analysis of video material showing the servicing activities undertaken by an employee and only a few of the listed works relate to real-time data analysis. Moreover, data were collected from an operational manufacturing cell without any guided or scripted work. Research experiments were conducted wherein we used three types of CNN network. The first network was based on resnet18 (from SVM) and was used to extract frame features. Frame features are one of the key elements in deciding whether the activity undertaken by a newly employed worker is correct. Next, it is necessary to check the classes of objects found on the frames of the video material. For this purpose, a combination of CNN network and R-CNN detector was used. In this case, the network was based on Cifar10Net architecture. For real-time image analysis, a model based on Yolov3 (COCO) was created. 

So, having reference material that properly presents servicing activities being carried out as they should be carried out, allows the key image frames, representing the stages of individual activities, to be determined. At the same time, data regarding objects in designated frames are recorded. During the training of a new employee, the employee is filmed on an ongoing basis and the frames of the image of the material are recorded by the camera and analysed by the CNN network for similarity of features and the occurrence of objects. Only when there is a similarity of frame features and the set of objects in the reference and test frames match, does the model decide which action is currently being performed.

Experimental results illustrate that the deep learning approach proposed, is effective for recognising tasks in an operational manufacturing cell. However, the following restrictions have been noted:

- in 13.64% of cases, the network repeatedly found objects of the same class.
- in relation to test frames, in 42.5% of cases, the network did not find such objects as Cuvette, Motoreductor, Wrench or Hand. The reason for this is probably the overlap of objects on the frames of the video material, the size of the objects not matching the size of the objects in the set on the basis of which the CNN network was taught and finally the learning set being too small.

Eliminating these restrictions will constitute ongoing research work for the authors, which will consist, first of all, in increasing the teaching set on the basis of which the convolution network was trained.

The proposed model (CNN + R-CNN and four new algorithms) can be applied, firstly, to automatically learn how activities at the workplace should be carried out and secondly, in order to automatically detect mistakes in the activities carried out by workers at the workplace. This is particularly useful in the process of training and for checking the skills and abilities of new employees in the company. The video can be used to provide workers with direct visual feedback and whether they are at the appropriate stage of work and therefore whether they are able to be trained and able to learn how their activities should be undertaken. 

The two-stage process implemented, excluded the detection of activities based solely on the similarity of frame features. Despite high compatibility, no object on the frame required activities to be carried out; detection was based on objects appearing on the image, despite the fact that the classes of elements on the reference image and the overlapping test frame characteristics were too dissimilar. The main limitation of the proposed work is that detecting the tools on the frames does not mean that the employee uses them correctly. This process should be accompanied by some other assessments detecting whether tools are being used correctly. In further work, the rules specifying the duration of each activity were formulated and added. Moreover, further work involves analysing the location of individual objects on the frames of video material. 

Moreover, in further work, there is a need to focus on recognising actions that simultaneously accommodate many workers, contained in the video frames, within the whole production process. We have developed efficient and robust deep learning algorithms that analyse received data and which recognise worker activity; in this way we will focus our research on increasing the reference material and improving our deep learning algorithms.

## 6. Conclusions

This paper presents the results of research in the field of improving the processes of verification of the automation of employee work in manufacturing companies. The model proposed renders controllable the activities, carried out by the newly employed worker, by comparing the video input with the activities carried out alongside the reference material, thanks to the connection implemented: CNN, R-CNN and four, newly created algorithms.

The main advantage of the proposed approach is the two-stage, checking and comparison of the video material received, against the reference material; this ensures accuracy in the detection of mistakes in the activities carried out by the workers and confirms the validity of the innovative model proposed. Our idea was to create a model for verifying the correctness of the activities undertaken by a new employee in his/her work, namely, the repair of a solid-fuel boiler. 

We believe, that supervising and controlling this work can be done automatically, having registered video material, containing expert examples of the way a particular job should be done by a specialist.

## Figures and Tables

**Table 1:** New contributions of the approach to comparing the proposed approach with the closest related models in HAR, as applied to manufacturing.

Reference	Methods	Applied to HAR	Applied to Manufacturing	Applied to the Automated Assessment of Worker Activity in Manufacturing
[12]	CNN + LSTM	yes	no	no
[28,36]	CNN + SVM	No	yes	no
[35]	SAX	No	yes	no
[40]	CNN + DTW	yes	no	no
[43]	CNN + LSTM	yes	no	no
[3]	KNN, NB and SVM, a decision tree, a random forest classifier and a gradient boosting classifier CNN	yes	no	no
[21]	CNN + LSTM	yes	no	no
[25]	RNN + Gated Recurrent Unit	yes	no	no
[22]	Markov models, Naive Bayes, K-means	yes	yes	no
This paper	CNN, CNN + SVM, R-CNN	yes	yes	yes

**Table 2:** Activities and reference frames associated with each stage of the activity.

ACTIVITY	FRAME NUMBER
[1] STOPPING THE SOLID FUEL BOILER	[1,1]
	[1,2]
	[1,3]
[2] UNSCREWING THE MOTOREDUCTOR SCREWS	[2,1]
	[2,2]
	[2,3]
[3] REMOVING THE CLEANER	[3,1]
	[3,2]
	[3,3]
	[3,4]
[4] REMOVING THE MOTOREDUCTOR WITH THE AUGER	[4,1]
	[4,2]
	[4,3]
	[4,4]
[5] ASSEMBLING THE AUGER AND THE MOTOREDUCTOR	[5,1]
	[5,2]
	[5,3]
[6] TURNING THE MOTOREDUCTOR MOUNTING SCREWS	[6,1]
	[6,2]
	[6,3]
[7] INSTALLING THE CLEANER	[7,1]
	[7,2]
	[7,3]

**Table 3:** Results of Algorithms 4 and 5.

ACTIVITY	STAGE	FLAG	NUMBER OF TEST FRAME	DIFFERENCE OF FRAMES
0	0	1	1	7.60 × 10^−^^7^
1	1	1	2	8.33 × 10^−^^7^
1	2	1	103	7.48
1	3	1	241	8.33 × 10^−^^7^
0	0	1	256	7.60 × 10^−^^7^
2	1	1	269	9.75
2	2	1	331	1.09 × 10^−^^6^
2	3	1	349	1.11 × 10^−^^6^
0	0	1	411	7.60 × 10^−^^7^
3	1	1	433	1.11 × 10^−^^6^
3	2	1	567	1.15 × 10^−^^6^
3	3	1	593	9.81
3	4	1	815	8.23
0	0	1	912	7.60 × 10^−^^7^
4	1	1	978	9.75
4	2	1	1065	9.42
4	3	1	1144	8.83
4	4	1	1316	8.69
0	0	1	1321	7.60 × 10^−^^7^
5	1	1	1322	9.85
5	2	1	1436	8.41
5	3	1	1506	7.60 × 10^−^^7^
0	0	1	1509	8.33 × 10^−^^7^
6	1	1	1520	7.48
6	2	1	1576	8.33 × 10^−^^7^
6	3	1	1599	7.60 × 10^−^^7^
0	0	1	1619	9.75
7	1	1	1623	1.09 × 10^−^^6^
7	2	1	1722	1.11 × 10^−^^6^
0	0	1	1727	7.60 × 10^−^^7^

**Table 4:** Result of the evaluation of the model.

ACTIVITY	STAGE	FLAG	NUMBER OF TEST FRAME	DIFFERENCE OF FRAMES	COMPATIBILITY IN%
0	0	1	1	7.60 × 10^−^^7^	~100%
1	1	1	2	8.33 × 10^−^^7^	~100%
1	2	1	103	7.48	85.49
1	3	1	241	8.33 × 10^−^^7^	~100%
0	0	1	256	7.60 × 10^−^^7^	~100%
2	1	1	269	9.75	84.89
2	2	1	331	1.09 × 10^−6^	~100%
2	3	1	349	1.11 × 10^−6^	~100%
0	0	1	411	7.60 × 10^−^^7^	~100%
3	1	1	433	1.11 × 10^−^^6^	~100%
3	2	1	567	1.15 × 10^−6^	~100%
3	3	1	593	8.83	86.48
3	4	1	815	8.69	86.93
0	0	1	912	7.60 × 10^−^^7^	~100%
4	1	1	978	9.85	84.69
4	2	1	1065	8.41	87.63
4	3	1	1144	1.08 × 10^−6^	~100%
4	4	1	1316	7.60 × 10^−^^7^	~100%
0	0	1	1321	9.62	85.89
5	1	1	1322	9.25	86.06
5	2	1	1436	1.13 × 10^−6^	~100%
5	3	1	1506	7.59 × 10^−^^7^	~100%
0	0	1	1509	1.16 × 10^−^^6^	~100%
6	1	1	1520	8.31	87.71
6	2	1	1576	4.86 × 10^−5^	99.99
6	3	1	1599	8.83	86.48
0	0	1	1619	8.69	86.93
7	1	1	1623	7.60 × 10^−^^7^	~100%
7	2	1	1722	9.85	84.69
0	0	1	1727	8.41	87.63

**Table 5:** Table comparing Resnet18 and AlexNet networks (the comparison concerned the values of features of one video material.

NETWORK	ACCURACY%
Resnet18	~94%
Alexnet	~87.51%

**Table 6:** Table with comparison of Cifar10Net and AlexNet networks (test carried out on 60 frames—30 standard, 30 random).

NETWORK	ACCURACY%
Cifar10Net	~61.66%
Alexnet	~48.33%

**Table 7:** Table with comparison of Cifar10Net, AlexNet and YOLOv3 networks.

NETWORK	ACCURACY%
Cifar10Net	~61.66%
Alexnet	~48.33%
YOLOv3	~73.15%

**Table 8:** Comparison of the effectiveness of using CNN, CNN + R-CNN for identifying the stage of activity in the model for verifying the skills of a new employee in carrying out servicing.

	CNN	ACTIVITY DETECTION	CNN	(R-CNN) OBJECT DETECTION	ACTIVITY DETECTION
	95				
**COMPATIBILITY IN%**		YES	95	YES	YES
			
	86.3				
		YES	86.3	NO	NO
			

**Table 9:** The comparison results with existing, state-of-the-art methods.

Paper	Methods	Dataset	Type of Detection	Accuracy (%)
[12]	CNN + LSTM	own dataset	Safe actions	97
[12]	CNN + LSTM	own dataset	Unsafe actions	92
				
[28]	CNN + SVM	ImageNet	Overall	93.44
[36]	CNN + SVM	Bearing vibration dataset, Case Western Reserve University	ISAX based features	88
[5]	CNN, RNN	own dataset		97
[43]	CNN + LSTM	Market-1501, CUHK03, DukeMTMC-reID	Identification + Triplet loss	72.96
[43]	CNN + LSTM	Market-1501, CUHK03, DukeMTMC-reID	f_c_	67
[43]	CNN + LSTM	Market-1501, CUHK03, DukeMTMC-reID	f_m_	69.82
[21]	CNN + LSTM	DEAP dataset	2 (Hv/Lv))	84.16
[21]	CNN + LSTM	DEAP dataset	SEED-dataset 3	90.81
[25]	RNN + Gated Recurrent Unit	UCF Sports, UCF101, KTH	KTH (GMM + KF)	71.1
[25]	RNN + Gated Recurrent Unit	UCF Sports, UCF101, KTH	KTH (GRNN)	86
[25]	RNN + Gated Recurrent Unit	UCF Sports, UCF101, KTH	KTH (GMM + KF + GRNN)	96.30
[25]	RNN + Gated Recurrent Unit	UCF Sports, UCF101, KTH	UCF Sport (GMM + KF + GRNN)	89.01
[25]	RNN + Gated Recurrent Unit	UCF Sports, UCF101, KTH	UCF 101 (GMM + KF + GRNN)	89.30
[22]	Markov models, Naive Bayes, K-means	own dataset	point-by-point	70
[22]	Markov models, Naive Bayes, K-means	own dataset	point-by-point with FSHMM	65.8
This paper	CNN, CNN + SVM	own dataset		94.01
This paper	YOLOv3	own dataset		73.15
